# The phytochemicals and health benefits of *Cyclocarya paliurus* (Batalin) Iljinskaja

**DOI:** 10.3389/fnut.2023.1158158

**Published:** 2023-04-06

**Authors:** Yingbin Shen, Yao Peng, Xucheng Zhu, Haimei Li, Liwen Zhang, Fanlei Kong, Jia Wang, Di Yu

**Affiliations:** ^1^School of Life Sciences, Guangzhou University, Guangzhou, China; ^2^School of Pharmacy, Guangxi University of Chinese Medicine, Nanning, Guangxi, China

**Keywords:** *Cyclocarya paliurus*, nutritional composition, extraction methods, biological activities, functional applications

## Abstract

*Cyclocarya paliurus* (*C. paliurus*), a nutritional and nutraceutical resource for human and animal diets, has been constantly explored. The available biological components of *C. paliurus* were triterpenoids, polysaccharides, and flavonoids. Recent studies in phytochemical-phytochemistry; pharmacological-pharmacology has shown that *C. paliurus* performed medicinal value, such as antihypertensive, antioxidant, anticancer, antimicrobial, anti-inflammatory and immunological activities. Furthermore, *C. paliurus* and its extracts added to drinks would help to prevent and mitigate chronic diseases. This review provides an overview of the nutritional composition and functional applications of *C. paliurus*, summarizing the research progress on the extraction methods, structural characteristics, and biological activities. Therefore, it may be a promising candidate for developing functional ingredients in traditional Chinese medicine. However, a more profound understanding of its active compounds and active mechanisms through which they perform biological activities is required. As a result, the plant needs further investigation *in vitro* and *in vivo*.

## 1. Introduction

With the improvement of modern life quality and the rapid development of science and technology, more and more diseases of modern civilization come along with it. These diseases include cardiovascular and cerebrovascular diseases, hypertension, hyperglycemia, hyperlipidemia, etc. (1–3). Plants provide the wealthiest resource of functional natural compounds, which directly or indirectly aroused a wide range of applications for the well-being of the human population and domestic animals (4). With global attention to health, there is more and more research on preventing and treating civilized diseases. Many herbal plants with health benefits have been developed, and *C. paliurus* is one of them.

**Figure 1:**
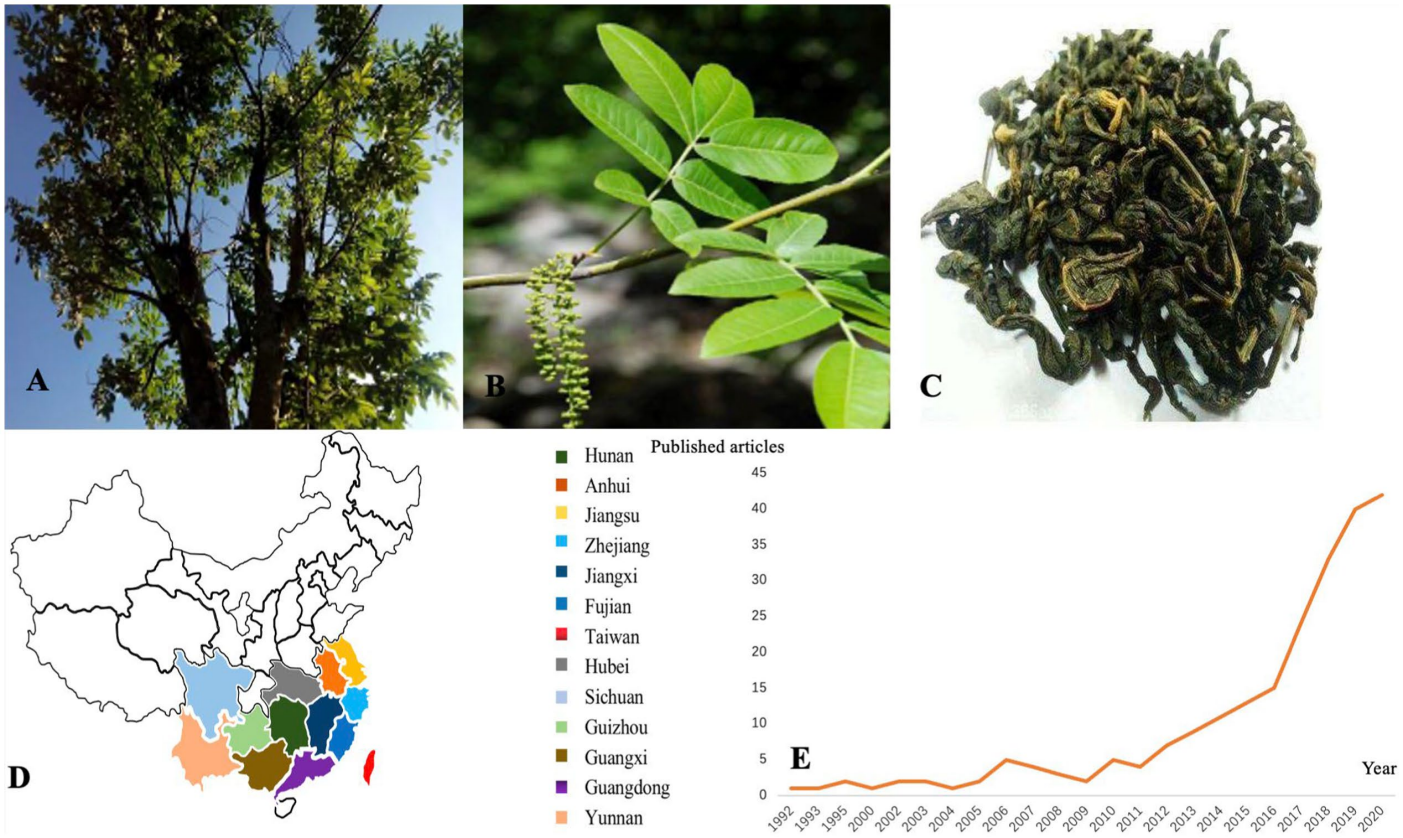
**(A)** *Cyclocarya paliurus* tree; **(B)** *C. paliurus* leaves; **(C)** *C. paliurus* tea; **(D)** *C. paliurus* distribution in China; **(E)** Publication of *C. paliurus* articles from 1992 to 2020.

*Cyclocarya paliurus* (Batalin) Iljinskaja, as shown in Figures 1A–C, is a deciduous tree belonging to the genus *Cyclocarya Iljinskaja* (Juglangdaceae) (5), which is called “sweet tea” in China. As a nutritional and nutraceutical resource for human and animal diets, *C. paliurus* is widely distributed in south China, such as Hunan, Hubei, Sichuan, Guangdong, and other southern cities (6). The medicinal virtues of *C. paliurus* have been well-known for their uses in several diseases and disorders since ancient times. As a traditional Chinese medicine, *C. paliurus* contains rich biological active substances, such as flavonoids, polysaccharides, triterpenic acids, and other essential human trace elements (7–9). In addition, *C. paliurus* leaves have been widely used as a therapeutic drug in traditional Chinese medicine to treat hyperlipidemia, coronary heart disease and hyperglycemia or to improve immunity (10–13). In 2013, it was approved as a new food ingredient by the National Health and Family Planning Commission of China (14). Recently, people have used *C. paliurus* leaves for tea because of their diverse biological and physiological activities (15). *Cyclocarya paliurus* tea became the first health tea approved by the Food and Drug Administration (FDA) in China in 1999 Due to its potential to develop healthy food which can treat diseases, improve work efficiency, and help people recover from mental fatigue (16). According to the Web of Science database statistics, the research articles about *C. paliurus* have been recorded since 1992 with 2,778 cited frequencies, as shown in Figure 1E.

*Cyclocarya paliurus* has a long history in China, and its active components and physiological functions are gradually being developed. However, there needs to be a systematic description of the chemical composition, bioactivity, and applications of *C. paliurus*. In order to elucidate the importance of *C. paliurus* to human health, we discussed its phytochemical composition and health effects through the previous research results. This review also aims to provide researchers with background and the latest research progress. This review analyzes the problems and development prospects of *C. paliurus* research and provides a scientific basis for utilization and further development.

## 2. Phytochemicals types

### 2.1. Polysaccharides

*Cyclocarya paliurus* polysaccharides (CP) is a bioactive component mixture with various physiological functional ingredients, which has aroused great interest and widespread concern (17, 18). As a result, more research has been done on developing CP and corresponding functional foods in recent years. Extraction of plant polysaccharides requires pretreatment of raw materials, such as washing, drying, grinding, sieving, degreasing with petroleum ether, and then refluxing with 80% ethanol to remove various impurities, such as monosaccharides, disaccharides, glycosides, polyphenols, etc. Numerous studies have shown that the polysaccharides of *C. paliurus* are composed of arabinose, glucose, xylose, mannose, rhamnose, and galactose (19–21). Currently, the mainstream methods for extracting CP are the traditional hot water extraction method, ultrasonic-assisted extraction method (UAE), and microwave-assisted extraction method. Yang et al. extracted polysaccharides from *C. paliurus* using distilled water as the extraction solvent. The optimum conditions include extraction temperature of 100°C, extraction time of 4 h, a material/water ratio of 1:20 and two rounds of extraction, which provide a final crude polysaccharides yield of 4.37% (22). In another study, ultrasonic-assisted extraction, microwave-assisted extraction, and heat reflux extraction (HRE) were used to compare the extraction rates of CP. The results showed that the ideal extraction technique is microwave-assisted extraction, which had the highest yield of polysaccharides (5.07 ± 0.02%), and the HRE method and UAE method were 3.89 ± 0.04% and 4.82 ± 0.03%, respectively (23). The extracted crude polysaccharides were then precipitated, deproteinated and dialyzed, followed by column purification with DEAE-Sephadex A-25 column, AB-8 macroporous anion-exchange column, DEAE-cellulose chromatography, and D301R chromatography column (22, 24–26). The structural characteristics of the polysaccharides of *C. paliurus* are shown in Table 1.

**Table 1:** Polysaccharides isolated from *C. paliurus*.

Compound	Source	Yield (%)	Monosaccharide composition	MW (kDa)	Extraction technique	Chain structure	Bioactive	Ref
CPP	Jiangxi, China		Rhamnose, arabinose, xylose, mannose, glucose, and galactose in the molar ratio of 1.00:2.23:0.64:0.49:0.63:4.16	Two fractions, with average MWs of 1.35 × 10^5^ Da and 9.34 × 10^3^ Da	Hot water extraction		Anti-hyperlipidemic	(22)
CPP	Jiangxi, China		Rhamnose, arabinose, xylose, mannose, glucose and galactose in the percent of 10.6, 30.2, 4.6, 8.7, 31.8, 14.1%.		Hot water extraction			(27)
CP, UCP	Jiangxi, China		Arabinose, galactose, glucose and galacturonic acid in the molar ratio of 1.0:4.6:3.5:4.5 Arabinose, galactose, glucose, Galacturonic acid in the molar ratio of 1.0:5.9:3.9:4.4	1.36 × 10^3^ 1.34 × 10^3^	Hot water extraction ultrasonic-assisted extraction		Antioxidant	(19)
CPP-1	Jiangxi, China		Xylose, arabinose, glucose, galactose, rhamnose and mannose in a molar ratio of 1.00:9.67:9.65:4.96:3.29:2.70	1,167 kDa	Hot water extraction		Antioxidant	(24)
CPP-D	Jiangxi, China		Mannose, glucose and galactose in a molar ratio of Man, Glc, Gal in a molar ratio of 0.235:0.677:0.088	9.1× 10^3^ Da	Hot water extraction	→4)-β-D-Glc-(1→, →2,6)-β-D-Man-(1→, and→4)-β-D-Gal	Antioxidant	(26)
CPPS-A CPPS-B CPPS-C CPPS-D	Jiangxi, China			>300 kDa, 100–300 kDa; 6–100 kDa; <6 kDa	Hot water extraction			(28)
CPP	Jiangxi, China	4.5%	Glucose, rhamnose, arabinose, xylose, mannose and galactose, with molar percentages of 32.7, 9.33, 30.6, 3.48, 10.4, and 13.5%.	900 kDa	Hot water extraction			(21)
PCP	Hunan, China	6.27%			Hot water extraction		Fatigue-alleviating effect	(16)
CPP-2	Jiangxi, China	4.37%.	Rhamnose, mannose, glucose and galactose in a molar ratio of 1.00:0.78:3.22:0.45	Two fractions, with average MWs of 1.35 × 10^5^ Da and 9.34 × 10^3^ Da	Hot water extraction		Anti-hyperlipidemic activity	(12)
CPP-3	Jiangxi, China		Rha, Ara, Xyl, Man, Glu and Gal in a molar ratio of 0.060: 0.109: 0.053: 0.128: 0.293: 0.357	Two fractions, with average MWs of 5.69 × 104 and 4.94 × 10^3^ Da	Hot water extraction		Inflammatory effects	(29)
CPP	Jiangxi, China		Rhamnose, arabinose, xylose, mannose, glucose, and galactose with a molar percentage of 3.39, 23.82, 6.98, 7.15, 17.79, and 40.87%.	Two fractions, with average MWs of 190.1 and 2.1 kDa	Hot water extraction		Lipid-lowering effect	(20)
CPP	Jiangxi, China		Rhamnose, arabinose, galactose, glucose, Mannose and xylose in a molar ratio of 1.00:0.78:3.22:0.45:1.00:1.85:3.26:3.12:0.85:0.29					(25)

**Figure 2:**
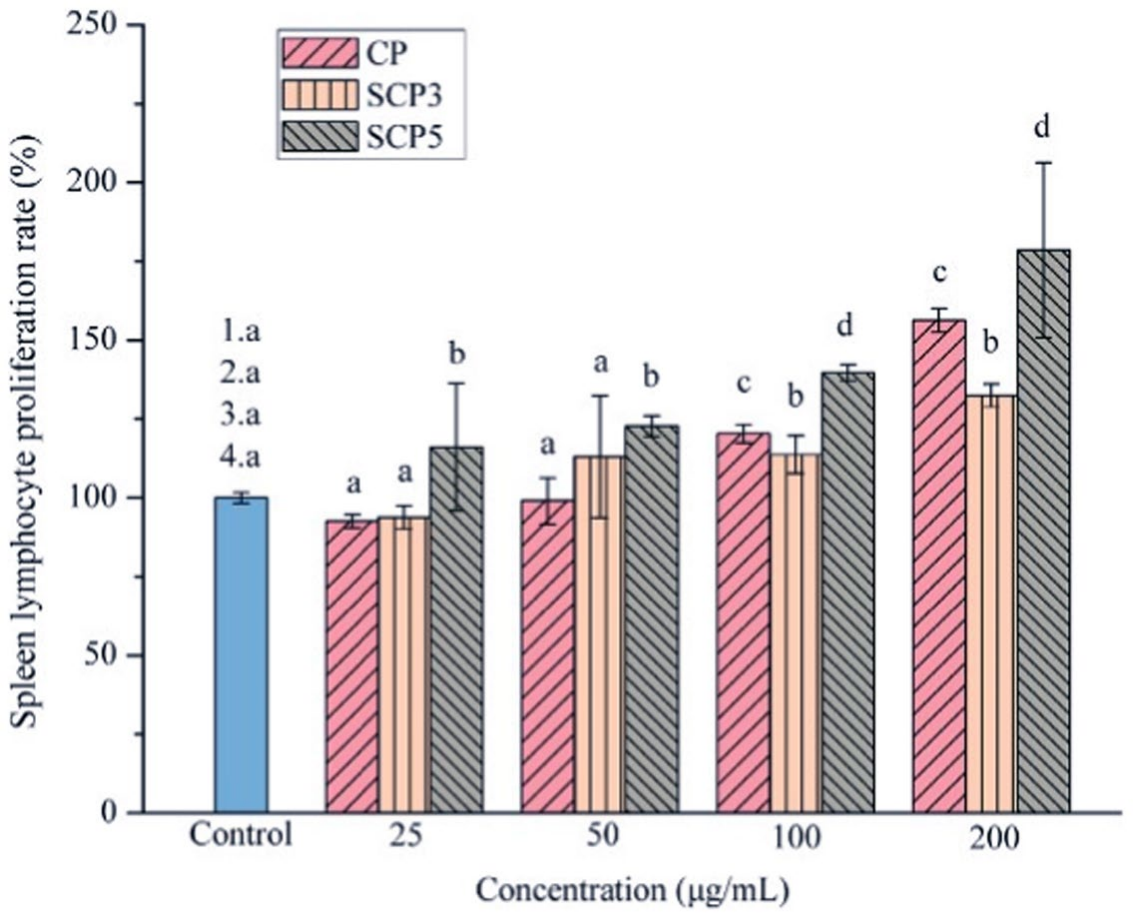
The effect of sulfate modification on CPP’s immunomodulatory enhancement (31).

Several factors would determine the biological activity of polysaccharides, such as the structure, molecular weight, functional groups, the sugar unit of the backbone, the type and polymerization degree of the branch, the glycosidic bond, and the conformation of polysaccharides (29, 30). Some modifications, such as sulfation, carboxymethylation, and acetylation, would change its biological activity. The modification of *C. paliurus* polysaccharides was then studied in different ways. Xie et al. extracted polysaccharides from *C. paliurus* and chemically modified them to obtain sulphated polysaccharides(S-CP). They found that DS (degree of substitution), Mw (molecular weight), sulfate content, protein, and uric acid content would be the main factors affecting the sulfated polysaccharides’ antioxidant activity. It was found that the physicochemical properties, monosaccharide composition, molar ratio, molecular weight, and immunomodulatory activity of the polysaccharides were changed after the sulfate modification (31). According to Yu’s research (Figure 2), SCP5(with a DS of 0.13) had better immunomodulatory activity than SCP3(with a DS of 0.45). So, we can conclude that a proper degree of sulfation may enhance the immunomodulatory activity of CCPs in a dose-dependent way. Among these sulfates, polysaccharides with different DS, S-CP_1–4_ (0.42 ± 0.04) and S-CP_1–8_ (0.06 ± 0.01) with relatively low DS had a better antioxidant effect, and their antioxidant activity was significantly enhanced with increasing concentration (19). Besides, based on the H_2_O_2_-induced RAW264.7 cells models, S-CP_1–4_ (400 μg/mL) treatment increased superoxide dismutase (SOD) activity was 1.29 ± 0.20 units, with CP (1.12 ± 0.18 units) and S-CP_1–8_ (1.08 ± 0.11 units). In addition, compared to S-CP_1–4_ and CP, S-CP_1–8_ performed a significantly higher antioxidant capacity (*p* < 0.05) and inhibited lipid oxidation (11). Another study through further in-depth chemical characterization revealed that S-CP_1–8_ consisted of Ara, Rha, Gal, Glc, Xyl, Man, GalA, and GlcA with a molar ratio of 1.0:0.1:1.8:1.1:0.2:0.6:0.4:0.1 and its molecular weight (Mw) was 970 kDa. Compared with CP, S-CP_1–8_ had immune-enhancing effects by significantly increasing the secretion levels of cytokines (TNF-α (Tumor Necrosis Factor-α), IL-1β (Interleukin-1β) and IL-6 (Interleukin-6)) in a dose-dependent manner, and enhancing Nitric Oxide (NO) release and phagocytic activity of macrophages (32). In addition, they successfully prepared the acetylated *C. paliurus* polysaccharides (Ac-CP). They found that its monosaccharide composition was Ara, Gal, Glc, Rha, Man and GalA with a molar ratio of 1.00: 1.67: 1.07: 0.15: 0.34: 1.58: 0.1, and its molecular weight was 1.05 × 10^6^ Da. Compared with the total polysaccharides, Ac-CP stimulated the production of pro-inflammatory cytokines by RAW264.7 cells. Acetylation modification could further enhance its activity to improve the immune activity of CP (33). Furthermore, they found acetyl groups were substituted at the O-2 and O-6 positions of 3)-β-D-Galp-(1 residue of the acetylated *C. paliurus* polysaccharides (Ac-CPP_0.1_). Ac-CPP_0.1_ treatment significantly increased the levels of SOD, Glutathione peroxidase (GSH-Px), and catalase in H_2_O_2_-treated dendritic cells. The molecular-level study revealed that the mechanism might be related to the activation of the Nrf2-Keap1 signaling pathway (34). Similarly, carboxymethylated *C. paliurus* polysaccharides (CM-CPs) affected the content of carbohydrates, proteins, and glyoxylates and changed the Mw and the molar ratio of monosaccharides. The result showed that the CM-CPs had higher ratios of Gal, Rha, and Glc and relatively high DS and Mw compared with native polysaccharides (35).

### 2.2. Triterpenoid saponins

Triterpenoid saponins are composed of oleanolic acid, ursolic acid, maslinic acid, corosolic acid, 23-hydroxyursolic acid, arjunolic acid, echinocystic acid, and other organic acids (36). Triterpenoid saponins are widely distributed in plants, such as *Glycyrrhiza uralensis Fisch*, *Ilex pubescens Hook*, *Panax ginseng*, and *C. paliurus*, among which *C. paliurus* has specific triterpenoid saponins. However, it has a few kinds. Different sources of triterpenoid saponins exhibit different bio functions, such as anticancer activity, anti-AIDS activity, hypoglycemic activity, and anti-cardiovascular activity. However, their mechanisms depend on the antioxidant activity resulting from the isoprene structure. A previous study found that ethanol extract of *C. paliurus* leaves was abundant in triterpenic acids (37). With different eluents, 13 saponins were obtained in *C. paliurus* leaves. Shu et al. isolated two secodammarane triterpenoid saponins from *C. paliurus* leave (38). Another study isolated new triterpenoid saponins from the ethanol extract of *C. paliurus*, and the structure was identified by spectroscopy (39)_._ Similarly, Shang et al. successfully quantified four triterpenoids in the leaves of *C. paliurus* by HPLC (40).

### 2.3. Polyphenols/flavonoids

The polyphenols in *C. paliurus* are extracted with a 1% hydrochloric acid solution. There are mainly two polyphenols (catechin and procyanidin B1) in *C. paliurus*. From 21 natural populations, the total polyphenol content of *C. paliurus* was 20.80–52.69 mg/g using the Folin–Ciocalteu, suggesting high phenolic content and antioxidant capacity may come from the low-temperature pressure (41, 42). Flavonoids are a type of polyphenol which exist in most plants. In recent years, the research on flavonoids of *C. paliurus* has increased gradually, which can provide a theoretical reference for developing potential value. Many flavonoids, such as isoquercetin, quercetin, and kaempferol, were identified from *C. paliurus* (7, 43) (Table 2). The extraction of flavonoids from *C. paliurus* included ethanol extraction (44), ultrasonic extraction (47), Enzymolysis-ultrasonic assisted extraction (43) (Table 3), and purification using AB-8 resin (7), HPD-600 macroporous resin (43), anion exchange, Sephadex G-100, and Sepharose CL-6B column. Liquid Chromatograph Mass Spectrometer (LC–MS) (17), GC, and high-performance liquid chromatography (HPLC) (43), ultra-performance liquid chromatography-quadrupole-time of flight mass spectrometry (UPLC-Q-TOF-MS) (44) are commonly used to identify flavonoids.

**Table 2:** Chemical constituents isolated from *C. paliurus*.

Main class	Sub-class	Name	Chemical structure	Ref
Flavonoids	Flavonols	Isoquercitrin	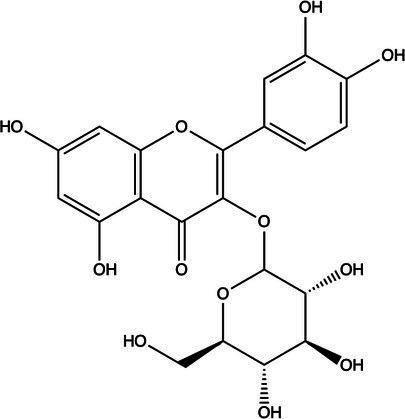	(7)
		Quercetin-3-O-rhamnoside	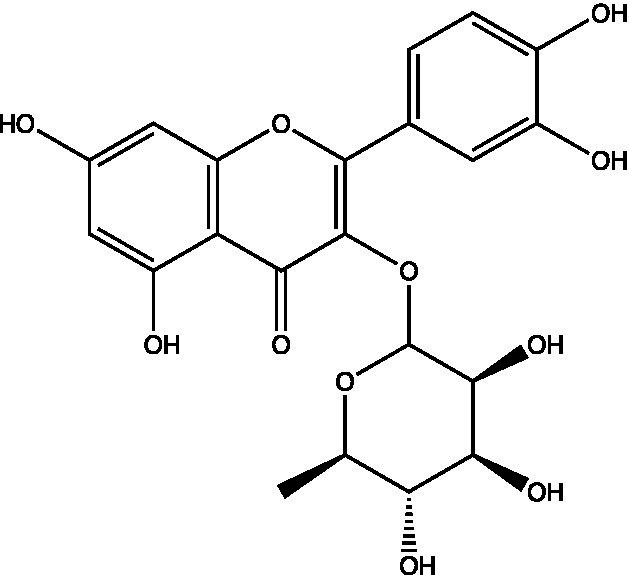	(44)
		Quercetin-3-O-glucuronide	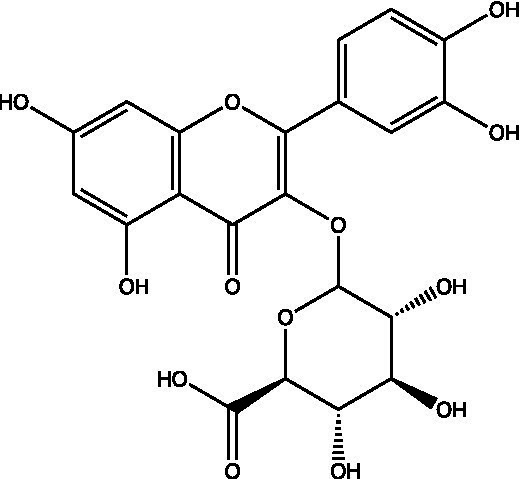	(45)
		Kaempferol-3-O-rhamnoside	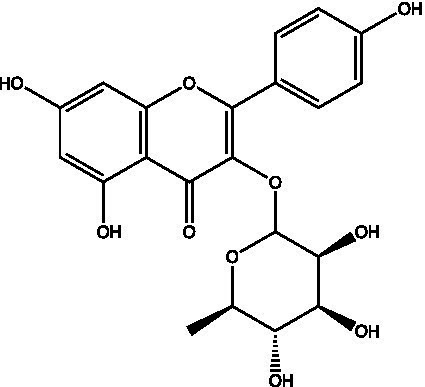	(45)
		Kaempferol-3-O-glucuronide	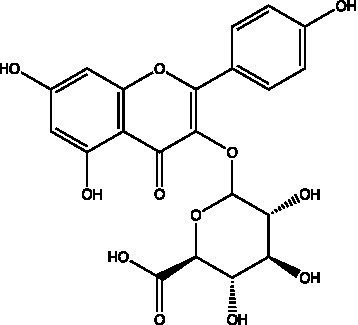	(45)
		Quercetin	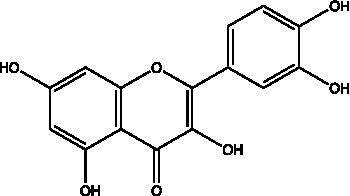	(44)
		Kaempferol	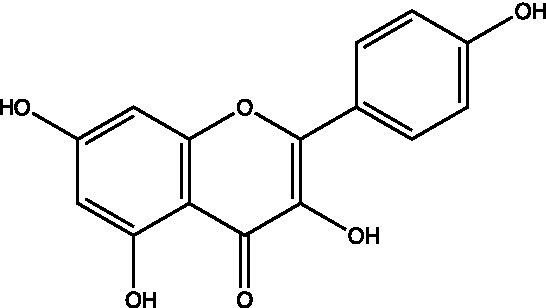	(44)
		Myricetin	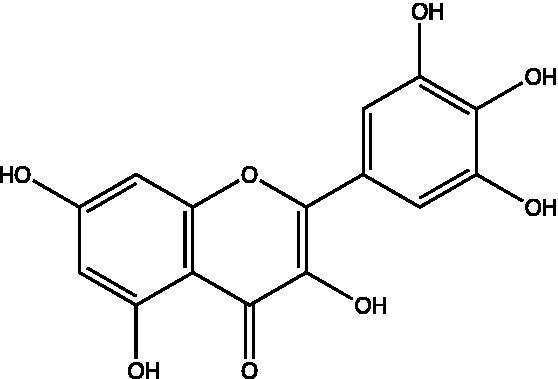	(46)
		Galangin	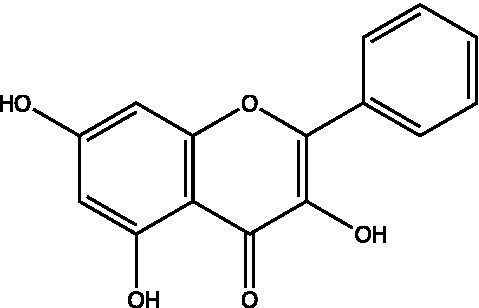	(46)
	Flavones	Luteolin	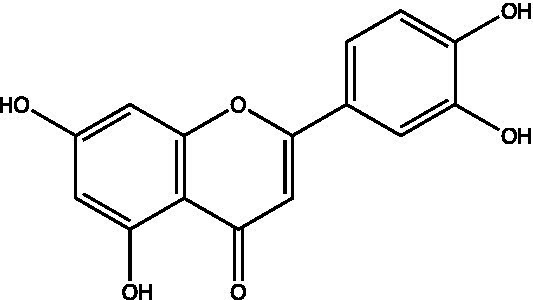	(21)
		Apigenin	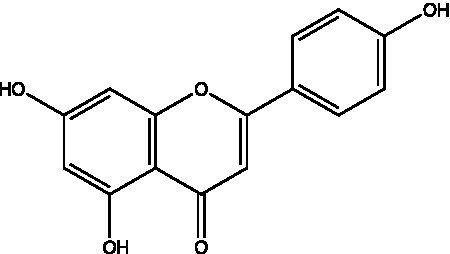	(21)
		Chrysin	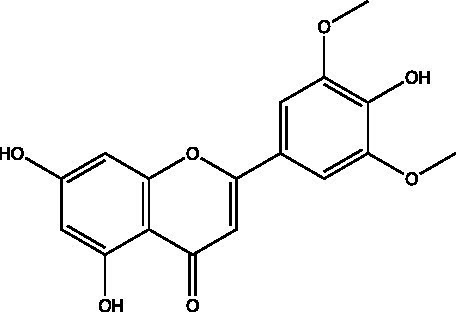	(21)
		3′, 4′-hydroxyflavanone	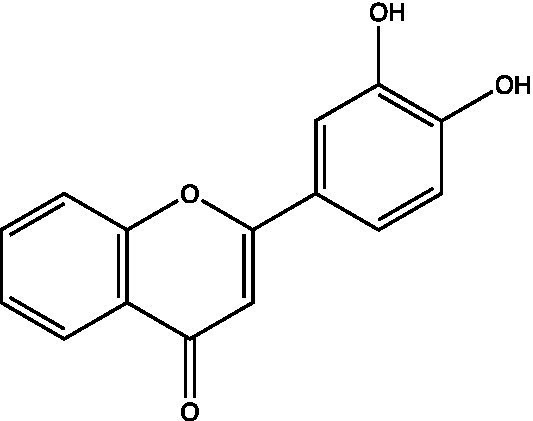	(21)
	Isoflavones	Daidzein	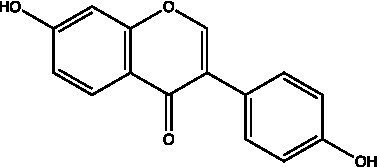	(21)
		Puerarin		(21)
		Biochanin A	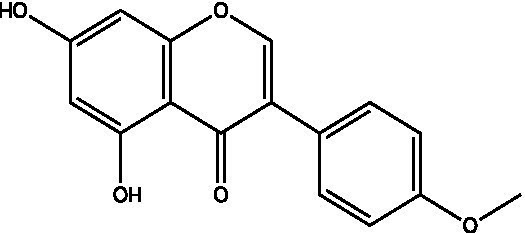	(21)
		Genistein	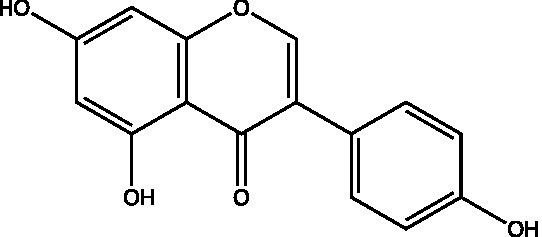	(21)
	Flavanones	3′-hydroxyflavanone	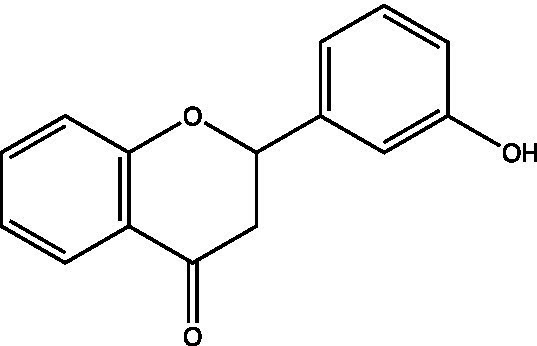	(21)
		4′-hydroxyflavanone	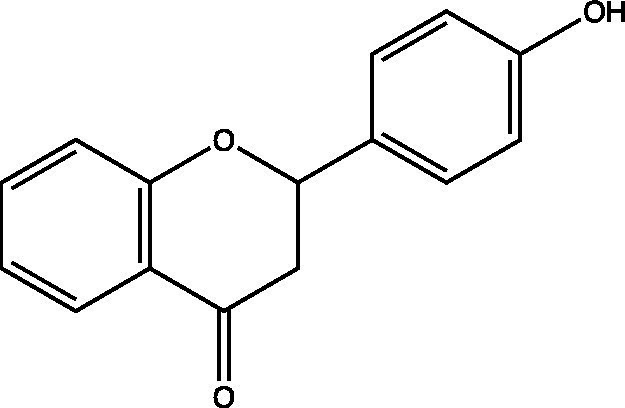	(21)
		Hesperetin	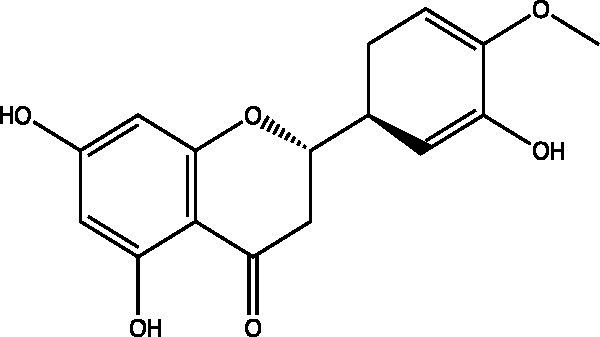	(21)
		Glycyrrhizin	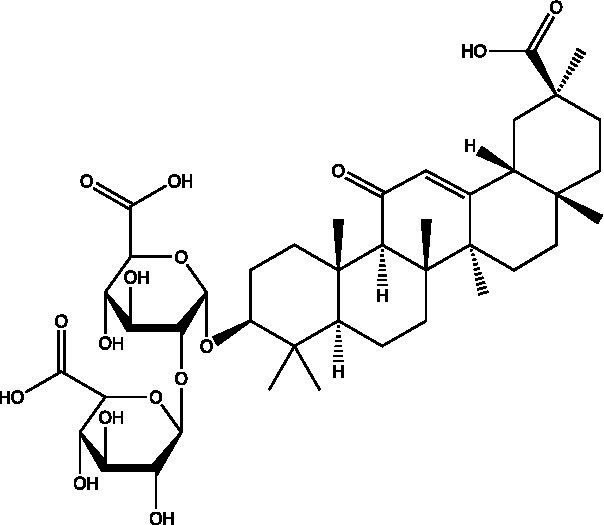	(21)
		Naringenin	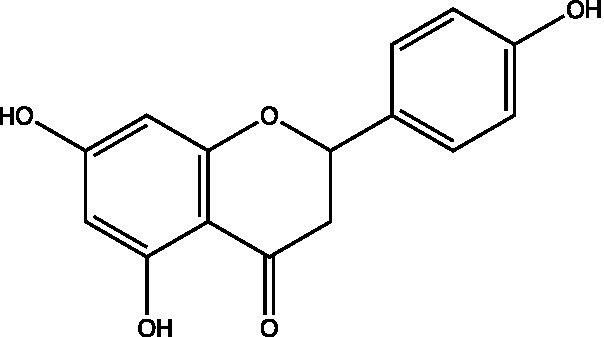	(21)
	Flavanonols	Dihydrokaempferol	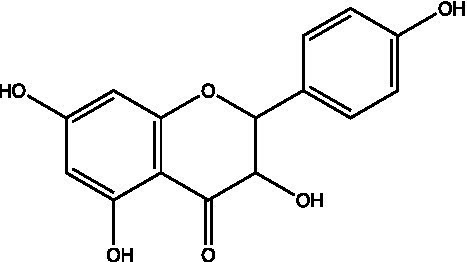	(21)
		Taxifolin		(21)
		Dihydromorin	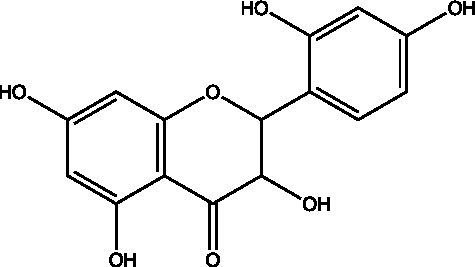	(21)
		Astilbin	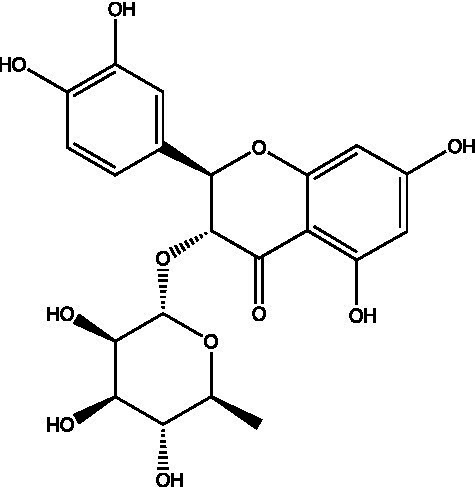	(21)

**Table 3:** A summary of the methods of extraction used for flavonoids from the leave of *C. paliurus*.

Extraction method	Extraction principle	Characteristic	Ref
Organic solvent extraction	Similar miscibility	Organic solvents are costly and potentially harmful	(48)
Alkali extraction	Flavonoids are easily soluble in alkaline water, but precipitate out under acidic conditions	Simple and easy	(49)
Supercritical fluid extraction	In the supercritical state, the solubility changes due to the pressure change to achieve the purpose of extraction	Large investment and high cost	(37)
Microwave assisted extraction	Utilizing the biological effect of microwave on cell membrane to improve the extraction rate	Fast extraction speed and high extraction rate	(35)
Enzymatic extraction	Enzymatic hydrolysis reaction by adding suitable enzymes to increase the yield of flavonoids	High catalytic efficiency, but may produce new substances, affecting purity	(40)
Ultrasonic assisted extraction	Destructive effect on cell membrane is conducive to the dissolution of flavonoids	Simple equipment and high extraction rate	(28)
Enzymolysis-ultrasonic assisted extraction	Through the destruction of cell wall by enzyme, the flavonoids in cell wall are released, so as to improve the yield of flavonoids. Ultrasonic cavitation effect and strong vibration can promote the release of flavonoids	Reduce production costs and accelerate the dissolution of flavonoids	(37)

Hu et al. used the colorimetric method to determine the total flavonoid content of CPF (*C. paliurus* flavonoids), which was calculated to be 752.60 ± 10.64 mg/g. Afterwards, using the UPLC-Q-TOF-MS analysis found CPF contained five main flavonoids, including quercetin-3-O-glycoside (47.38 ± 2.32 mg/g), quercetin-3-O-rhamnoside (38.83 ± 2.02 mg/g), kaempferol-3-O-rhamnoside (315.42 ± 12.23 mg/g), quercetin (97.23 ± 3.89 mg/g) and kaempferol(52.78 ± 3.21 mg/g) (44). Similar results were obtained by Lei et al. (43). Different purification methods and concentrations of ethanol solutions result in different flavonoid compositions and contents in *C. paliurus*. A study found that seven flavonoids of *C. paliurus* leaves were composed of 3-O-caffeoylquinic acid (0.04–1.38 mg/g), quercetin-3-O-glucuronide (0.30–2.38 mg/g), kaempferol-3-O-glucuronide (0.17–1.88 mg/g), kaempferol-3-O-rhamnoside (0.05–2.30 mg/g), arjunolic acid (0.02–2.70 mg/g), cyclocaric acid B (0.04–0.98 mg/g), pterocaryoside B (0.01–2.46 mg/g), and pterocaryoside A (1.07–2.48 mg/g) (45). Another study found that Kaempferol-3-*O*-glucuronide was the main flavonoid in *C. paliurus* leaves, and the content of kaempferol-3-*O*-rhamnoside was 256.82 ± 9.53 mg/g, Kaempferol-3-O-𝛽-glucuronide was 472.47 ± 11.79, Isoquercitrin was 151.47 ± 3.74 mg/g, and Quercetin was 86.33 ± 1.55 mg/g (7). It is reported quercetin-3*-O-*b-D-glucuronide, quercetin, kaempferol-3-*O*-b-D-glucuronide, kaempferol-7-*O*-a-L-rhamnoside and kaempferol were main constituents in the *C. paliurus* leaves using LC–MS (17). It is also reported that Kaempferol-3-*O*-glucuronide was the main flavonoid in *C. paliurus* leaves whose content was 472.47 ± 11.79 mg/g. Meanwhile, the content of kaempferol-3-*O*-rhamnoside was 256.82 ± 9.53 mg/g, the content of Isoquercitrin was 151.47 ± 3.74 mg/g, and the content of Quercetin was 86.33 ± 1.55 mg/g (7). By comparing the adsorption effects of several macroporous resins and polyamide for separating flavonoids from *C. paliurus*, the influence of light and province on the flavone content is different in diverse regions. The flavone content in *C. paliurus* is also different due to the diversity of light and temperature (50).

## 3. Health benefits

The biological activity and many health properties of *C. paliurus* have been the focus of research *in vitro* and *in vivo*. As a result, there is a growing interest in the commercial application of *C. paliurus*. However, evidence from human epidemiological and interventional studies is still lacking. It is reported that *C. Palinuru*s’s biological activities include antioxidant, hypoglycaemic, hypotensive, anti-inflammatory, antitumour, anti-ageing, cardioprotective effects, etc.

### 3.1. Antioxidant activity

The antioxidant activities of *C. paliurus* have been widely studied. The extract of *C. paliurus*, such as polysaccharides, flavonoids, and triterpenoid saponins, are closely correlated with apparent antioxidant effects. Several studies have focused on the antioxidant activity of *C. paliurus*, and their results are presented in Table 4. The trace elements and vitamin E in *C. paliurus* also help prevent oxidation. For example, selenium is involved in the formation of glutathione peroxidase in the body, which scavenges excess free radicals in the body (17). Xie et al. evaluated the antioxidant activity of CPP, compared with the DPPH free radical scavenging rate of BHT (91.2%) and ascorbic acid (98.9%). The results showed that CPP was 91.4% (24). After the antioxidant activity of CPP has been purified with a further step by DEAE-Cellulose, it can also significantly reduce MDA (Malondialdehyde) levels, increase the activity of SOD (Superoxide dismutase), T-AOC (Total antioxidant capacity,) and CAT (Catalase) (26). The antioxidant activity of CPP differs by different extraction methods. Research shows that ultrasonic treatment can improve the antioxidant activity of CPP by changing the monosaccharide composition. The difference may be caused by the change in monosaccharide composition, but the relationship between the enhancement of antioxidant activity and the change needs further investigation (19).

**Table 4:** Antioxidant activities of *C. paliurus*.

Extraction	Dose	Antioxidant activity	Ref.
CPP	50–400 μg/ml	DPPH radicals: 47.6–91.4% EC_50_: 52.4 μg/ ml	(24)
CP, UCP	31.3–500 μg/ ml 500–1,000 μg/ml 62.5–500 μg/ml	DPPH radicals:40.9 ± 2.2%→90 reactive oxygen radicals:31.7 ± 2.5–87.2 ± 2.6% -Carotene-linoleic acid assay:17.5 ± 4.9% -59.0 ± 2.2%	(19)
CM-CPs	Superoxide radicals: CM-CP1:0.5 mg/mL CM-CP2:0.5 mg/mL CM-CP3:0.5 mg/mL hydroxyl radical: CM-CP1:0.5 mg/mL CM-CP2:0.5 mg/mL CM-CP3:0.5 mg/mL -Carotene-linoleic acid assay: CM-CP1:1.0 mg/mL CM-CP2:1.0 mg/mL CM-CP3:1.0 mg/mL	Superoxide radicals CM-CP1:47.26 ± 1.53% CM-CP2:42.51 ± 0.89% CM-CP3:40.73 ± 1.32% hydroxyl radicals: CM-CP1:73.23 ± 0.58%, CM-CP2:61.97 ± 0.76%, CM-CP3: 73.75 ± 0.95% -Carotene-linoleic acid assay: CM-CP1:82.16 ± 1.04% CM-CP2:81.07 ± 0.68% CM-CP3:94.13 ± 1.28%	(35)
S-CP	S-CP_1–4_: 0.25 mg/mL S-CP_1–8_: 0.25 mg/mL	DPPH free radicals: S-CP_1–4_:87.02 ± 1.52% S-CP_1–8_:88.09 ± 1.62%	(14)
Ac-CPP_0.1_, CPP_0.1_	DPPH free radicals: Ac-CPP_0.1_:4.0 mg/mL CPP_0.1_:4.0 mg/mL ABTS radicals: Ac-CPP_0.1_:4.0 mg/mL CPP_0.1_:4.0 mg/mL	DPPH free radicals: Ac-CPP_0.1_:91.29 ± 1.30% IC_50_:0.42 mg/mL CPP_0.1_:82.61 ± 2.19% IC_50_:0.47 mg/mL ABTS radicals: Ac-CPP_0.1_:90.65 ± 1.32% IC50:0.82 mg/mL CPP_0.1_:72.35 ± 1.67% IC_50_:0.50 mg/mL	(11)
CPP, CPP-D	SOD: CPP: 25 μg/ ml CPP-D: 50 μg/ ml	SOD: CPP:3.68 U/mL CPP-D:3.80 U/mgprot	(26)
P-CP	DPPH:0.25 mg/mL hydroxyl radicals: 0.5 mg/ml	DPPH free radicals: 73.28 ± 0.41% hydroxyl radicals: 57.1 ± 0.18%	(51)
Phenolic Compounds		DPPH IC_50_ mg/mL: 3-*O*-caffffeoylquinic acid: 24 ± 0.003 4-*O*-caffffeoylquinic acid: 0.17 ± 0.001 4,5-di-*O*-caffffeoylquinic acid: 0.24 ± 0.002 Quercetin-3-*O*-glucuronide: 0. 12 ± 0.000 Quercetin-3-*O*-galactoside: 0.13 ± 0.002 Quercetin-3-*O*-glucoside: 0.13 ± 0.002 Quercetin-3-*O*-rhamnoside: 0.13 ± 0.000 Quercetin: 0.008 ± 0.000 Kaempferol: 0. 26 ± 0.002 FRAP TEAC/g: 3-O-caffeoylquinic acid: 3.51 ± 0.02 4-O-caffeoylquinic acid: 3.38 ± 0.06 4,5-di-O-caffeoylquinic acid: 4.70 ± 0.04 Quercetin-3-O-glucuronide: 3.77 ± 0.02 Quercetin-3-O-galactoside: 3.33 ± 0.01 Quercetin-3-O-glucoside: 2.80 ± 0.03	
		Quercetin-3-O-rhamnoside: 2.65 ± 0.02 Kaempferol-3-O-glucuronide: 0.22 ± 0.00 Kaempferol-3-O-glucoside: 0.30 ± 0.0 Kaempferol-3-O-rhamnoside: 0.30 ± 0.03 Quercetin: 11.25 ± 0.24 Kaempferol: 3.36 ± 0.05 ABTS TEAC/g: 3-O-caffeoylquinic acid: 4.58 ± 0.03 4-O-caffeoylquinic acid: 4.91 ± 0.07 4,5-di-O-caffeoylquinic acid: 5.13 ± 0.03 Quercetin-3-O-glucuronide: 4.44 ± 0.04 Quercetin-3-O-galactoside: 4.41 ± 0.01 Quercetin-3-O-glucoside: 4.27 ± 0.06 Quercetin-3-O-rhamnoside: 4.39 ± 0.06 Kaempferol-3-O-glucuronide: 0.55 ± 0.01 Kaempferol-3-O-glucoside: 0.57 ± 0.00 Kaempferol-3-O-rhamnoside: 0.68 ± 0.04 Quercetin: 6.37 ± 0.21 Kaempferol: 3.67 ± 0.09	(51)
MAE extracts	DPPH: 0.8 mg/mL	DPPH free radicals:94.68 ± 0.88% IC50: 0.146 mg/ml	(17)
Purified CPF	DPPH free radicals: 0.05–0.15 mg/mL	DPPH free radicals:56.57 ± 0.45%-93.06 ± 0.63%.	(43)
	Superoxide radicals: 0.05–0.15 mg/ml ABTS radicals: 0.05–0.25 mg/ml	Superoxide radicals:35.60 ± 0.53%–61.58 ± 0.46% ABTS radicals: 17.98 ± 0.54%–63.17 ± 0.85%	

Moreover, the derivatization of CPP can improve antioxidant function, including sulfation, phosphorylation, carboxymethylation, and acetylation. In the carotenoid-linoleic acid assay, the highest substituted carboxymethylated CPP showed high antioxidant activity. However, the ability to scavenge hydroxyl and superoxide radicals decreased (35). Sulfated CPP scavenged hydroxyl radicals in a dose-dependent manner. Among them, sulfate groups are considered to play an important role. The antioxidant activity of these sulfated polysaccharides may depend on the sulfated derivatives’ chemical composition, molecular weight, and specific structural units (14). Further, an oxidative stress model was prepared in the RAW264.7 cell line using hydrogen peroxide (H_2_O_2_). Compared with unmodified CPP, it can significantly enhance the proliferation and phagocytosis activity, inhibit lipid oxidation as determined by the level of MDA, and promote the production of nitric oxide (NO) in RAW264.7 cells, which suggests that the sulfated CPP has particular immunological activity (11). Similar experimental results were obtained for phosphorylated CPP. It can reduce oxidative stress and cell oxidative damage by increasing intracellular antioxidant enzyme SOD content and decreasing MDA content while decreasing apoptotic capacity, improving cell viability and achieving intracellular antioxidant activity. According to Xie’s research, phosphorylated CPP decreased the MDA content to 34.8% compared with the model group (51). Moreover, acetylated CPP achieves antioxidant activity by regulating the Nrf2-Keap1 signaling pathway and improving the activity of antioxidant enzymes (34).

The phenolic compounds of quercetin and kaempferol glycosides are the main antioxidant components in the ethanol extracts of palm leaves (52). The content of phenolic compounds in the extracts from the leaves of *C. paliurus* was positively correlated with antioxidant activity. Cao et al. found that extracts with higher phenolic compound content had higher antioxidant activity (53). The Zhou et al. study found that the total polyphenol contributed most significantly to the antioxidant activity of 15 natural populations among flavonoids, phenolics and triterpenoids (54). In addition, the antioxidant capacity of phenolic compounds was related to the number and configuration of H-donating hydroxyl groups (53). Xie et al. isolated and purified flavonoids from the leaves of *C. paliurus*. The result showed that the DPPH radical scavenging rate of CPF was lower than that of V_C_ but higher than that of BHT. The antioxidant activity of CPF was evaluated *in vitro* by DPPH radical-scavenging activity and showed higher antioxidant activity depending on the dose with higher concentrations (17). Xiong et al. extracted CPF by enzymatic digestion-assisted ultrasound. They employed three agents to analyze the antioxidant activity: DPPH radical scavenging activity, superoxide radical scavenging activity, and ABTS^+^ radical scavenging activity. To this end, Purified CPF showed significant scavenging activity against DPPH, superoxide and ABTS+ radicals, showing a dose-dependent scavenging activity (43). *In vivo* study was conducted on the beneficial effects of polysaccharides from the leaves of *C. paliurus*. It was found that CPP-treated worms had a robust antioxidant defense system, with an 81% reduction in ROS accumulation and reduced content of peroxidation products (MDA, NEFAs (Nonest esterified fatty acid) and GSSG (Glutathione)), while increasing antioxidant-active enzymes (SOD, CAT and GSH-Px (Glutathione peroxidase)) and GSH Glutathione, r-glutamyl cysteinyl ^+^glycine) (52). Lin et al. developed a beverage which contains *Momordica saponins* and CPP. The *in vivo* study showed that it could promote nuclear localization of DAF-16 and translation of SOD-3 to activate the antioxidant system, reduce the levels of ROS (Reactive oxygen species), MDA, and NEFAs, and alleviate age-related pigmentation and damage caused by neurodegenerative diseases. In contrast, inhibiting of fat accumulation provides antioxidant protection and reduces oxidative damage (55). CPP alleviates ROS-induced oxidative stress mechanisms *in vitro* and *in vivo*, possibly by enhancing the scavenging activity of antioxidant enzymes on liver and kidney free radicals and inhibiting CYP2E1 expression, thereby improving oxidative stability (8). Further studies revealed that induction analysis of stress resistance indicated that the CPP-mediated response CPP-mediated stress response is dependent on skn-1 and hsf-1, which may act together with genes responsible for stress induction, including sod-3, sod-5 hsp-16.1, hsp-16.2, ctl-1, and ctl-2 (18).

### 3.2. Hypoglycemic activities

The role of *C. paliurus* in regulating diabetes has also been demonstrated. Hyperglycemia is one of the main symptoms of diabetes. Due to the apparent hypoglycemic effect of *C. paliurus*, it has aroused broad interest among researchers. Several researchers have investigated the hypoglycemic activity of *C. paliurus* through *in vivo* studies (Table 5). Zhang et al. reported the hypoglycemic bioactivity of triterpenic acid of *C. paliurus* through *in vivo* and *in vitro* experiments. A kidney injury model was established *in vitro* using HG-induced HK-2 cells, and CPT treatment significantly ameliorated HG-induced apoptosis in kidney cells. *In vivo* studies showed that triterpenic acid with different concentrations significantly reduced blood glucose in STZ (Streptozocin)-induced diabetic rats and showed a significant increase in body weight.

**Table 5:** *In vivo* hypoglycemia experiments of *C. paliurus*.

Extraction	Experiment details	Result	Ref
CP ethanol extract (CPEE), CP aqueous extract (CPAE)	140 rats were fed with HFD and high sugar water for 6 weeks, then injected with 30 mg/kg STZ after 5 days. In addition, 100 HFD-STZ-induced diabetic rats were randomly divided into 10 groups: non-diabetic control group, diabetic control group, sample groups: treated with different doses of CPEE and CAEP (2,4,8 g/kg), positive control groups (250 mg/kg MHT and 1.73 g/kg XKP) with the determination of glucose metabolism, lipid metabolism, antioxidant activity, and renal function.	CPEE and CPAE had similar biological activities. CPEE had a lower polysaccharides content than CPAE. The enhancement of SOD activity by CPAE was significantly better than that of CPEE, suggesting that polysaccharides may be potential antioxidant components of CP. Both CPEE and CPAE increased OGTT (oral glucose tolerance test), insulin tolerance test (ITT), ITT, HDL-C, SOD, and GSH-Px, and decreased TC, TG, LDL-C, MDA, BUN (Blood urea nitrogen), and CREA (Creatinine), GSP.	(15)
The barks of *C.paliurus*	100 alloxan-induced diabetic rats were randomly divided into eight groups: standard control group, model control group, 0.15 g/kg positive drug metformin hydrochloride group, different dose sample treatment groups (0.25, 0.5, 1.0 g/kg Petroleum ether fraction; Chloroform fraction; Ethyl acetate fraction; n-Butanol fraction; Aqueous fraction) for 7 days.	Reducing blood glucose levels.	(56)
The aqueous extract of leaves of CP (ACP)	66 male Wistar albino rats were divided into 5 groups: standar1 control group, DN (Diabetic nephropathy) model group (fed with a high-fat diet for 6 weeks and injected with a dose of 35 mg/kg STZ), DN rats were treated with 0.047 and 0.094 g/kg ACP, DN rats were treated with 0.075 g/kg ascorbic acid.	ACP decreased blood glucose levels and renal AR activity, inhibited PP, reduced RI (renal index), BUN (blood urea nitrogen), SCr (serum creatinine), Upro (urine protein), Cysc (cystatin C), AMG levels (α1 microglobulin), pro-inflammatory cytokines IL-6 and ET-1, and attenuated renal injury.	(57)
CPF	The microbial community present in fresh fecal samples from 6 healthy volunteers (3 females and 3 males, 25–30 years old) was initially colonized in 6-week-old male C57BL/6 J mice by diluting freshly excreted human fecal samples (1 g) in 10 ml of reduced phosphate-buffered saline (0.1 mol/l, pH 7.2) and suspending the fecal material by vertexing up, and 0.2 ml of the suspension was introduced into each sterile receptor by gavage. Six-week-old male C57BL/6 J mice were acclimatized by feeding a high-fat diet for 7 days and then randomly divided into three groups of eight mice each: high-fat diet group (HFD), low-fat diet group (LFD, HFD plus CPF supplementation (HFD-CPF) group. In the HFD-CDF group, CPF was thoroughly mixed with HFD with a final concentration of 0.1% (w/w) for 8 weeks. For the HFD-CDF group, CPF was mixed with HFD to a final concentration of 0.1% (w/w). Stool samples were collected from the HFD-CPF group (CPF 0, CPF 2, CPF 4, and CPF 8).	CPF increased intestinal microbial diversity, regulated microbial composition, maintained microecological balance and regulated certain metabolic pathways.	(7)
CPE	ICR mice were injected with STZ (200 mg/kg), and normal group mice were injected with the same volume of saline. Mice injected with STZ were randomly divided into 2 groups, the control group (n = 8) and the CPE group (n = 9), with a dose of 1 g/kg in the CEP group, and blood samples were drawn at 0, 1 and 2 h after oral administration of CPE to measure blood glucose and insulin levels.	CPE given to mice decreased plasma glucose levels and increased the phosphorylation of AS160 and Akt in skeletal muscle.	(58)
CPT	Male Sprague–Dawley rats were injected with STZ (65 mg/kg) *via* intraperitoneal injection, and control rats were injected with the same volume of sodium citrate buffer (*n* = 12). STZ-induced rats were randomly divided into four groups (*n* = 12): Control group: given 0.5% sodium carboxymethylcellulose (10 ml/kg CMCeNa). STZ Group: given 0.5% CMCeNa. Met Group: treated with Metformin (200 mg/kg). Low-dose CPT Group (CPTL): treated with 40 mg/kg CPT. High dose CPT Group (CPTH): treated with 160 mg/kg CPT. All groups were treated with daily intragastric administration for 10 weeks.	CPT effectively decreased body glucose, reduced microalbumin, serum creatinine and blood urea nitrogen levels, and ameliorated the increase in thylakoid stroma and glomerular fibrosis. The hypoglycemic mechanism of CPT may be related to the AMPK-mTOR-regulated autophagy pathway while preventing kidney injury and apoptosis through autophagy.	(59)
The extract of *C. paliurus* leaves	Eight-week-old male C57/BL6J mice were fed with HFD for 4 weeks and injected with STZ, and control mice were injected with an equivalent amount of the drug. The STZ-induced mice were randomly divided into five groups: diabetic group (0.5% CMC-Na), glibenclamide-treated diabetic group (15 mg/kg/day), low/medium/high dose CPE treated diabetic groups (1, 2, 4 g/kg/day).	Decreasing the plasma levels of AST, ALT, TG, TCHO, LDL/VLDL. Decreasing the wet kidney/body weight ratio and reducing plasma CREA levels. Increasing the plasma HDL, reducing glomerular basement membrane thickening and plasma CREA levels and improving the pathological changes of nephropathy and cardiac hypertrophy. Exhibiting potential anti-hyperglycemic effects and protecting against diabetes-related complications.	(60)
CPE	Kunming male mice were administered CPE (100, 200, and 500 mg/kg and 200 mg/kg), orally.	CPE inhibited the overexpression of TNF-a, IL-6, MCP-1, resistin, and the inflammation-induced serine phosphorylation of IRS-1, restored the phosphorylation of IRS-1 on tyrosine residues and the phosphorylation of downstream Akt., and increased the expression of adiponectin in adipose tissue and promoted the glucose consumption of adipocytes.	(61)

In addition, significant urinary protein levels in the urine improved renal meningeal stromal accumulation and glomerular fibrosis. CPT (*C. paliurus* triterpenoids) treatment significantly increased the phosphorylation of AMPK (Adenosine 5′-monophosphate (AMP)-activated protein kinase) and decreased the phosphorylation of its downstream effector mTOR (mammalian target of rapamycin). It effectively improved kidney damage caused by diabetes (59). Xiao et al. found that CPE (CP extract) significantly reduced the proportion of apoptotic cells, inhibited apoptosis in pancreatic β-cells, and inhibited STZ-induced upregulation of caspase-3 and caspase-9 in a dose-dependent manner. CPE effectively inhibited the STZ-induced increase in ERK (extracellular regulated protein kinases), JNK (c-Jun N-terminal kinase), and p38 phosphorylation in NIT-1 cells and increased in a dose-dependent manner reduction of Akt (protein kinase B) phosphorylation. Bodyweight, food intake and plasma glucose levels were significantly decreased in mice treated with CPE. The wet pancreas/body weight ratio, plasma insulin levels, β-cell area, and insulin-secreting β-cells were significantly increased (60). These findings suggest that CPE protects pancreatic β-cells from apoptosis by affecting MAPK (mitogen-activated protein kinase) and Akt signaling pathways. Another related study found that CPE enhanced tyrosine phosphorylation of insulin receptor substrates in C2C12 cells and activated phosphatidylinositol 3-kinase and Akt *via* sirtuin1.

**Figure 3:**
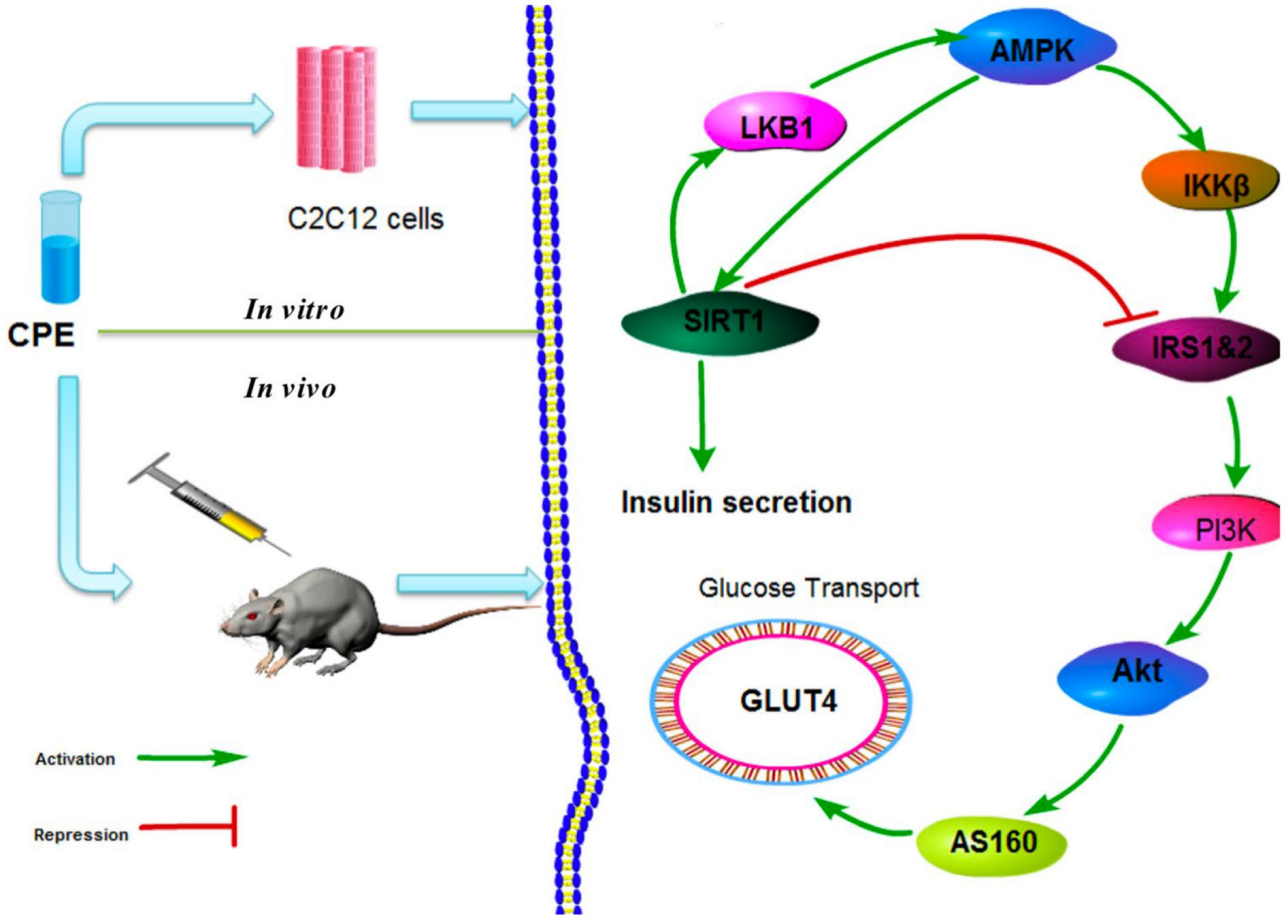
CPE *in vivo* and *in vitro* hypoglycemic mechanism (58).

Furthermore, oral administration of CPE increased the expression of Glut4 in skeletal muscle membranes. It enhanced the phosphorylation of Akt in STZ-induced hyperglycemic mice with reduced plasma glucose levels, and the signaling pathway is shown in Figure 3 (58). Jiang et al. also found that CPE can regulate adipokine expression and improve insulin resistance by suppressing inflammation in mice (61). Li et al. established a diabetes model. Their results showed that Various fractions of *C. paliurus* bark significantly reduced blood glucose levels in diabetic rats, especially the chloroform fraction (56). Their biological activities determined the phenolic compounds from *C. paliurus* leaves through PTP1B inhibition experiments. Compared with the control group (HD0518), the study found that the naphthoquinone derivatives and phenolic compounds in the extracts inhibit the activity of PTPIB as a potential functional food ingredient to prevent and treat diabetes and obesity (62).

### 3.3. Hypolipidemic activities

Several research groups have investigated the hypoglycemic activity of *C. paliurus* in animal models. The results are presented in Table 6. The hypoglycemic activity of CPP was investigated by high-fat emulsion (HFE)-induced hyperlipidemic rats for 8 weeks. The addition of CPP to the diet caused a significant decrease in plasma TC (total cholesterol), TG (Triglyceride), and LDL-C (Low-Density Lipoprotein Cholesterol) levels. It increased HDL-C (High-density Lipoprotein Cholesterol) levels in HFE diet rats. CPP significantly increased ATGL (Adipose Triglyceride Lipase) and PPAR (peroxisome proliferators-activated receptors) expression in the liver group, while hepatic FAS (Fatty acid synthase) and HMG-CoA (3-hydroxy-3-methyl glutaryl coenzyme A reductase) expression were relatively decreased (22). They demonstrated for the first time that CPP regulates the promoter expression levels of leptin and MTTP (Microsomal triglyceride transfer protein) by altering DNA methylation in the promoter (63). The team used CPP-2 purified by a DEAE-cellulose column as a raw material for further investigation. Similar experimental results were obtained. Compared with the model control group, CPP-2 significantly decreased TG, TC and LDL-C levels and increased HDL-C levels in mice. Also, CPP-2 significantly counteracted the increased oxidative stress by lowering LPO (Lipid peroxide) and MDA levels in serum and liver and increasing the activity of SOD, GSH-PX, GPT and T-AOC (12). While in another study, Lin et al. elucidated the lipid-lowering effect and mechanism of CPP on *Caenorhabditis elegans*. The result showed that CPP regulates lipid metabolic pathways through the MUFA biosynthetic pathway, mdt-15/sbp-1 and NHR-49/MDT-15 signaling pathways to reduce fat accumulation in *C. elegans* (64). Zhu et al. found that CPP can protect islets by reducing oxidative stress and pro-inflammatory cytokines. It also reduced dyslipidemia, hepatic steatosis, and liver diseases while CPP down-regulated lipid-induced metabolism, redox, and apoptosis-related genes and up-regulated miR-199a/31a gene, indicating it can help prevent liver damage (21). Yao et al. found that the preventive effect of CPE on hyperlipidemia and obesity was partly due to blocking fat absorption from the intestine by inhibiting the activity of apoB48. It is hypothesized that it may lower serum and liver cholesterol by inhibiting the activity of 3-hydroxy-3-methylglutaryl coenzyme A reductase while enhancing the activity of cholesterol 7α-hydroxylase to promote cholesterol excretion (10). In addition, triterpenoids were isolated from cyanobacteria and found to inhibit lipoprotein B48 secretion in the cells in a Caco-2 cell model (36).

**Table 6:** *In vivo* hypoglycemia experiments of *C. paliurus*.

Extraction	Experiment details	Result	Ref
CPP	Hyperlipidemic rats were given 20 mg/kg BW/d HFE for 8 weeks. CPP polysaccharides group: high-dose polysaccharides group (800 mg/kg BW/d), medium-dose polysaccharides group (400 mg/kg BW/d) and low-dose polysaccharides group (200 mg/kg BW/d). Model group: 400 mg/kg BW/d distilled water Simvastatin group: 20 mg/kg BW/d simvastatin.	Compared with the model group, CPP significantly reduced serum LDL-C, TG, and TC levels in hyperlipidemic rats, while HDL- C levels were significantly increased. In addition, it decreased FAS and HMG-CoA and upregulated the expression of ATGL and peroxisome-activated receptors.	(22).
CPP-2	Sixty female ICR mice were randomly divided into 6 groups of 10 mice each. The normal control group was perfused with 15 ml/kg body weight/day of distilled water by gastric tube. The experimental group was instilled with 15 ml/kg body weight/day of high-fat emulsion: the model control group was instilled with 15 ml/kg BW/d of distilled water. 50, 300 and 600 mg/kg BW/d were instilled in the CPP low-dose, medium-dose and high-dose groups, and, respectively, the positive control group was instilled with 5 ml/kg BW/d of simvastatin.	CPP-2 treatment improves blood lipid levels, liver lipid levels, and antioxidant status while reducing hepatocyte damage.	(12)
CPP	20 normal rats were fed with a standard diet as the regular group. In addition, 84 hyperlipidemic rats were randomly divided into 5 groups. The model group received an equal volume of normal saline orally), the positive group 10 mg/kg BW/d simvastatin, and the CPP low dose group 200 mg/kg BW/d intragastrically, the medium dose group 400 mg/kg bw/d intragastrically and the high dose group 800 mg/kg BW/d intragastrically for 8 weeks.	CPP significantly decreased abdominal wall adiposity index, liver weight, and serum concentrations of TC, TG, and LDL-C in mice. In contrast, HDL was increased. Also, it significantly downregulated DNA methylation levels and mRNA content of leptin, DNA methylation levels and mRNA content of MTTP.	(63)
CPE	Sprague Dawley (SD) rats were randomly divided into six groups of ten rats each. Normal control group (NC): fed with 10 ml/kg of distilled water daily. Other groups were fed a high-fat diet (HFD) after. Hyperlipidemic control group (HC): fed 10 ml/kg/day of distilled water. CPE group: fed orally with low, medium, and high doses of CPE (2, 4, and 8 g/kg). Green tea group (GT): fed green tea extract (2 g/kg) daily as a positive control.	CPE significantly reduced serum TC, TG, LDL-C, FFA, and leptin levels increased HDL-C and adiponectin levels, reduced body weight gain, lowered visceral fat weight, and reduced fat storage in rats.	(10)
CPP	The drugged normal group was incubated on NGM plates containing a mixture of *E. coli* OP50 (Ecoli OP50) and CPP at a concentration of 13.75 μg mL-1 (10 μl CPP stock solution added to 90 μl *E. coli* OP50)	CPP inhibited the effects of lipid accumulation in normal and high-fat *C. elegans*, altered the size distribution of lipid droplets, and reduced the number of lipid droplets.	(64)

### 3.4. Immunomodulatory activity

The inflammatory response is a defensive response of the body when it is invaded or infected by pathogens. Macrophages are important to the immune system members, and macrophages’ activation is a critical factor in the inflammatory response. Table 7 lists the immunological activities of the active substances of *C. paliurus*. CPP-3 was studied in the RAW264.7 cell assay and significantly promoted the release of TNF-α, IL-1 and PGE2 from cells when CPP-3 concentrations ranged from 12.5 to 200 μg/ mg (*p* < 0.01). In LPS -stimulated RAW264.7 cells, CPP-3 inhibited the cellular release of NO (Nitric Oxide), TNF-α, IL-1, and PGE2 (Prostaglandin E2) (*p* < 0.01) and had a synergistic effect on NO production (29). Meanwhile, CPP-3 up-regulated the expression levels of iNOS (Inducible Nitric Oxide Synthase), IFN-γ (Interferon-γ), TNF-α, IL-12 and IL-18 mRNA in RAW264.7 cells (65). Furthermore, modified CPP was non-toxic to RAW264.7 cells and enhanced the immunomodulatory effect on RAW264.7 macrophages compared to CPP (32, 33). Further studies have shown that the effect of sulfated CPP with the degree of substitution on cell proliferation is different. However, all of them can promote the secretion of Th1 cytokines, IL-2 and TNF-α, and Th2 cytokine IL-4 (31), which exerts immune mechanisms related to MAPK and NF-κB (nuclear factor kappa-B) pathways (66).

**Table 7:** Immunomodulatory activity of *C. paliurus*.

Extraction	Experiment details	Result	Ref
CPP-3	Pretreatment of RAW264.7 cells with different concentrations of CPP-3 (12.5, 25, 50, 100, 200 μg /mL) in the presence or absence of LPS (10 μg /mL) for 24 h	CPP-3 and LPS have synergistic effects on the release of NO and TNF-a and antagonistic effects on the release of IL-1 and PGE2.	(29)
CPP-3	Pretreatment of RAW264.7 cells with different concentrations of CPP-3 (12.5, 25, 50, 100, 200 g/ml) in the presence or absence of LPS (10 μg /mL) for 24 h	CPP-3 and LPS have synergistic effects on the release of NO and TNF-a and have antagonistic effects on the release of IL-1 and PGE2.	(65)
AC-CP	RAW264.7 cells were treated with 10 μg/ml of LPS as a positive control and 25, 50, 100 and 200 μg /ml of Ac-CP and CP.	Compared with unmodified CPP, AC-CP could significantly stimulate the proliferation of macrophages, stimulate the phagocytic activity of macrophages, and significantly enhance cytokines TNF-a, IL-1, and IL-6 levels. In addition, it indicated that the acetylated derivative AC-CP could enhance the activation of macrophages.	(33)
S-CP_1-8_	RAW264.7 cells were incubated with different concentrations of S-CP_1-8_ and CP at 12.5, 25, 50, 100, 200, and 400 μg /mL for 24 h.	Compared to CP, S-CP_1-8_ stimulates the activation of macrophage proliferation and NO production and increases the secretion of TNF-a, IL-1 and IL-6.	(32)
CPP, S-CPP, CPP_0.05_, S-CPP_0.05_	CPP, S-CPP, CPP_0.05_, and S-CPP_0.05_ were used to treat cells at 37.5, 75, and 150 μg/ml, and RPMI-1640 medium, and 10 μg/ml LPS were used as normal and positive controls, respectively.	Under the treatment of CPP and S-CPP, p-JNK, p-p38MAPK, and NF-κBp65 proteins were significantly increased, and blocking TLR2/4 could significantly decrease the above proteins.	(66)
SCP3, SCP5	The experiment was divided into a blank control group (100 μL/well RPMI-1640 complete medium); polysaccharides sample group (5, 50, 100, 200 μg/mL of CP and SCP,100 μL/well) and Concomplant ConA or LPS stimulation group (different concentrations of CP and SCP were added to each well) with final concentrations of 5 μg/mL and 10 μg/mL of ConA or LPS, respectively.	CP and SCPs stimulate the proliferation of splenic lymphocytes and enhance the immune response by secreting different cytokines, such as TNF-α, IL-2, and IL-4. SCP3 significantly stimulates the proliferation of T lymphocytes, while SCP5 significantly promotes the proliferation of B lymphocytes. In addition, SCP3 and SCP5 promote the secretion of the Th1-type cytokines IL-2 and TNF-α and the Th2-type cytokine IL-4 secretion.	(31)

### 3.5. Liver protection

Nowadays, liver injury caused by various chronic diseases has become one of the most common diseases in the world. Carbon tetrachloride (CCl_4_) is a typical environmental poison often used to establish animal models of acute and chronic liver and kidney diseases (67). Table 8 lists some studies on the hepatoprotective effects of *C. paliurus* substances. Xie et al. found that CPF significantly inhibited the increase of AST (Aspartate aminotransferase) and ALT (Alanine aminotransferase) activities in CCl_4_-induced liver injury serum in mice. Simultaneously, CPF would significantly reduce the content of MDA in liver tissues and enhance SOD and T-AOC activities in liver tissues (68). Wang et al. verified the protective effect of CPF against LPS/D-GalN-induced acute liver failure. The mechanism might be related to inhibiting NF-κB activation and activating the Nrf2/HO-1 pathway (44). Furthermore, CPT-regulated palmitic acid-induced hepatic steatosis in HepG2 cells showed that CPT could significantly reduce the number of lipid droplets and intracellular TG content PA-induced HepG2 cells and primary hepatocytes. The molecular mechanism studies showed that CPT regulates the pathway of the PI3K (phosphoinositide 3-kinase) /Akt/GSK3β (phosphorylation of glycogen synthase-3β). Similar experimental results were obtained in animal models, increasing PI3K, Akt and GSK3β, and the PI3K inhibitor was replaced by LY294002 (69). In another study, CP may protect the liver by improving oxidative stress to protect the liver and kidney from CCl_4_ damage (8). LPS (Lipopolysaccharide) stimulated the cells to secrete the opposite pro-inflammatory cytokines, and D-GalN (D-galactosamine) inhibited the mRNA synthesis of anti-apoptotic genes and induced liver necrosis.

**Table 8:** Liver protection of *C. paliurus*.

Extraction	Study	Model	Dose	Evaluated parameters	Result	Ref
CPF	*In vivo*	CCl_4_-induced Kunming mice	100, 500, 1,000 mg/kg	ALT, AST, TBA, CRE, enzyme activity, body weight, liver and spleen weight	CPF significantly reduced AST and ALT in the serum of CCl_4_-induced mice and significantly increased the liver’s T-AOC, GSH-Px, and SOD levels. Moreover, it reduced the index and weight of the liver and spleen.	(68)
*CPP*	*In vivo*	CCl_4_ mice	50, 100, 200 mg/kg bw/d	Body weight, relative organ index, ALT, AST, TBA, CRE, enzyme activity, SOD and GSH-Px	CPP ameliorated CCl_4_-induced weight loss and organ swelling in mice, reduced the levels of ALT, AST, TBA, and CRE in serum, and significantly decreased CYP2E1 expression and increased MDA, SOD, and GSH-Px levels in liver and kidney.	(8)
	*In vitro*	NCTC-1469 cell	50–1,000 μg/mL	GSH-Px, MDA, cell viability	CPP increased cellular viability and GSH-Px activity and reduced the production of intracellular MDA.	
CPF	*In vivo*	LPS/D-GalN-induced mice	100, 200, 400 mg/kg	Mice survival rate, ALT, AST, T-SOD, GHS-Px, immunohistochemistry analysis, histological examination、TNF-α、IL-1β、IL-6 、caspase-3、CD14、TNF-R1、Romo1、Bcl-XL、Nrf2、HO-1	CPF regulated the expression levels of Caspase-3, reactive oxygen species regulator 1 (romo1), and B-cell lymphoma super large cells (Bcl-Xl), downregulated the expression levels of CD14 and Tnf-R1, improved liver morphology and oxidative stress, and reduced the levels of ALT and AST and inflammatory cytokines.	(44)
CPT	*In vivo*	HFD-induced C57BL/6 J mice	40, 160 mg/kg	Food intake, body weight, Insulin sensitivity, TC, TG, glucose, ALT, AST, inflammatory cytokines.	CPT reversed liver injury in mice with NAFLD, reduced serum and liver lipid levels, and significantly inhibited the secretion of pro-inflammatory cytokines. Moreover, it significantly increased the expression of phosphorylated proteins of PI3K, Akt, and GSK3β.	(69)
	*In vitro*	HepG2 cells	0–100 μg/ml	cell viability, inflammatory cytokines.	CPT improved insulin resistance, had no significant effect on cell viability, inhibited intracellular lipid accumulation, reduced PA-induced hepatic steatosis, and increased IL-6, IL-1β, and TNF-α levels. At a CPT concentration of 25 μg/ml, the anti-inflammatory effect was almost equivalent to that of berberine (25 μg/ml).	

### 3.6. Other activities

In addition to antioxidant, hypoglycemic, and lipid-lowering effects, other aspects of *C. paliurus* are also being developed. Polysaccharides from *C. paliurus* leaves have antibacterial activity. It was found that CPP (1 mg/mL) had good antibacterial activity against Saccharomyces cerevisiae and Candida, and the inhibition zone was 9.72 ± 0.16 mm and 10.21 ± 0.37 mm, respectively. It also had specific antibacterial activity against *Escherichia coli*, *Staphylococcus aureus*, and *Bacillus subtilis*. The diameter of the inhibition zone was 6.54 ± 0.23, 6.57 ± 0.11, and 6.93 ± 0.45 mm, respectively. However, at the concentration of 1 mg/ml, the polysaccharides had no inhibitory effect on three kinds of fungi (Aspergillus niger, Mucor, and Penicillium) (23). In another study, the antibacterial activity of CPP was concentration-dependent. When the concentration of polysaccharides was 80 μg/ml, it had an apparent inhibitory effect on Staphylococcus aureus, Salmonella, Escherichia coli, and other microorganisms. The inhibition zone diameter was 21.5 ± 0.45, 17.5 ± 0.35, and 13.5 ± 0.25 mm, respectively. The antibacterial mechanism of CPP needs to be further studied. Experimental research by Lei et al. showed that when the concentration of flavonoids in *C. paliurus* is higher than 40 mg/mL, the antibacterial and bactericidal effects are better. The purified flavonoids in *C. paliurus* had the most substantial inhibitory ability against *Staphylococcus aureus*, followed by salmonella, and the weakest ability inhibitory capacity against *E.coli* (43). These results indicate that *C. paliurus* can be used as an antibacterial agent for some bacteria.

CPP has been proven to have antitumor activity, and its mechanism was explored by evaluating its antitumor activity *in vitro*. Different concentrations of CPP (50, 100, 200, and 400 μg/mL) were used to treat HeLa cancer cells. In the concentration range of 50–200 μg/mL with the increase of CPP concentration, the inhibitory effect on HeLa cells was significantly enhanced in a dose-dependent manner. The results showed that CPP could directly inhibit the proliferation of HeLa cancer cells, suggesting that CPP can inhibit the growth of HeLa cancer cells through S-phase cell cycle arrest and induce apoptosis (70). Moreover, the team continued to study CPP’s effect on the composition of intestinal flora. The results showed that CPP could significantly increase the short-chain fatty acids (acetic acid, propionic acid, butyric acid, and valeric acid) in the faeces of healthy mice in a dose-dependent manner.

Furthermore, 16S rRNA showed that CPP could effectively increase the diversity of intestinal microflora in healthy mice and affect the relative abundance of Chlamydia, Clostridium and Clostridium. In addition, PICRUSt2 showed that the phenotype of high-dose mice was metabolized by the KEGG pathway (48). Thus, CPP can enhance the metabolic function of intestinal microbiota by increasing the release of SCFAs and changing the composition of intestinal microbiota.

## 4. Conclusion and commercial value

This mini-review summarizes the extraction techniques, chemical structures, and biological activities of some of the polysaccharides, flavonoids, and saponins found in *C. paliurus*, suggesting that *C. paliurus* has potential medicinal and dietary value. In the last few years, *C. paliurus* has gained the attention of the scientific and business communities due to its versatility in food, pharmaceuticals, waste management, and sustainable agricultural practices. More recently, research interest has shifted toward incorporating *C. paliurus* trees into beverages and beverage products to improve physicochemical properties, shelf life, and nutritional value. Jiangxi Xiushui has developed a health drink from *C. paliurus*, which uses *C. paliurus* leaves as primary raw material for functional species of health drinks such as hypoglycemia, and is the first health tea to receive FDA quality approval. Beijing Orient Kewen has also developed a series of health products from *C. paliurus*, including tablets, granules, capsules, and lozenges, all of which have received the “Health Food Approval Certificate” issued by the state. In addition, the *C. paliurus* leaves are ground into flour and mixed with wheat flour to make biscuits. Compared with ordinary biscuits, *C. paliurus* biscuits have a hypoglycemic effect and suit people prone to obesity and high blood sugar. *C. paliurus* has a unique sweet flavour and is commonly used in folklore for sweet biscuits and sweets and is a natural food additive that can be added directly to food. Its main components are caryophyllene A and nucleoside I, which are more than 200 times sweeter than sucrose.

*Cyclocarya paliurus* is a plant with multidimensional utility and versatile applications in human nutrition. There is still a need for in-depth research into *C. paliurus*, through *in vivo* studies in animals and epidemiological studies, to better understand the effects on human health. *Cyclocarya paliurus* can be an excellent reserve for pharmaceutical and herbal preparations. The wealth of phytochemical information and advances in biotechnology have led to the creation of new avenues aimed at increasing the overall commercial value of the tree.

## Author contributions

JW and DY proposed the concept of the review. YS, YP, XZ, HL, and FK collected and analyzed the literature. YS, YP, and JW drafted the manuscript. JW, YS, DY, and LZ critically revised the manuscript. All authors have read and approved the final version of the manuscript.

## Conflict of interest

The authors declare that the research was conducted in the absence of any commercial or financial relationships that could be construed as a potential conflict of interest.

## Publisher’s note

All claims expressed in this article are solely those of the authors and do not necessarily represent those of their affiliated organizations, or those of the publisher, the editors and the reviewers. Any product that may be evaluated in this article, or claim that may be made by its manufacturer, is not guaranteed or endorsed by the publisher.

## Funding

This work was supported by China Postdoctoral Science Foundation (621312-2), Talent Project of Guangzhou University (Nos RP2020078 and RD2020052), the National Natural Science Foundation of China (32060576) and ‘Gui-pai Xinglin young talent’ from Guangxi University of Chinese Medicine (2022C026).
